# Primary sarcomatoid carcinoma of the trachea: A case report and literature review

**DOI:** 10.1097/MD.0000000000040766

**Published:** 2024-12-13

**Authors:** Xiuwen Yu, Mingqi Huang, Haiyan Ge, Junjie Yang, Bin Huang, Jialing Xu

**Affiliations:** a Department of Respiratory Medicine, The First People’s Hospital of Xiaoshan District, Hangzhou, Zhejiang, China; b Department of Traditional Chinese Medicine, Faculty of Chinese Medicine Science Guangxi University of Chinese Medicine, Nanning, China; c Department of Pathology, The Second People’s Hospital of Xiaoshan District, Xiaoshan District, Hangzhou, Zhejiang, China; d Department of Pathology, The First People’s Hospital of Xiaoshan District, Hangzhou, Zhejiang, China; e Departments of Medical Oncology, The First People’s Hospital of Xiaoshan District, Hangzhou, Zhejiang, China.

**Keywords:** clinicopathology, sarcomatoid carcinoma, trachea

## Abstract

**Rationale:**

Primary sarcomatoid carcinoma of the trachea (PSCT) is a rare malignant tumor of the lower respiratory tract. Pathological types of tracheal sarcomatoid carcinoma (TSC)s include pleomorphic carcinomas, giant cell carcinomas, spindle cell carcinomas, pulmonoblastomas, and carcinosarcomas. At present, there are limited reports on PSCT, and pathologists lack sufficient knowledge about it.

**Patient concerns:**

Here, we report a case of malignant neoplasm involving the left posterior wall of the initial tracheal segment, characterized by atypical spindle cells and a small number of high-grade squamous intraepithelial neoplasia.

**Diagnosis:**

Spindle-shaped cells were moderately heterotypic, with 8/10 nuclear divisions (high-magnification field), and no obvious inflammatory cell infiltration was observed in the interstitium. Squamous epithelial cells showed moderate-to-severe atypia with regional cytoplasmic transparency. Immunohistochemistry: Fusiform tumor cells expressed vimentin, epithelial markers were negative, the Ki-67 proliferation index was 40%, and epithelial markers were expressed in squamous intraepithelial neoplasia.

**Interventions:**

After the biopsy diagnosis was confirmed, part of the tumor was removed by tracheoscopy under general anesthesia in the respiratory department of a superior hospital. A pathological diagnosis of TSC was made and local radiotherapy was performed.

**Outcomes:**

As the tumor could not be completely cured, the patient experienced repeated coughing and shortness of breath and died of the disease 15 months later.

**Lessons:**

Pathological morphology and immunohistochemical analyses deepen our understanding of the pathological features of TSC and provide a diagnostic reference for clinicians who will encounter such cases in the future.

## 1. Introduction

Primary tracheal malignancies are uncommon, accounting for approximately 0.2% of all malignancies in adults.^[1]^ Experts have reported squamous cell carcinoma as the predominant histological type, accounting for 45% of cases, followed by adenoid cystic carcinoma,^[2]^ and several other types. Tracheal sarcomatoid carcinoma (TSC) is a rare type of non-small cell carcinoma. Since the first report by Aksu et al in 2009, only 6 cases have been reported in the literature.^[3–8]^ The clinical manifestations of TSC include dyspnea, cough, sputum production, wheezing, and other symptoms that are difficult to distinguish from those of asthma and chronic obstructive pulmonary disease. Computed tomography (CT) examination can reveal a mass at the tracheal site. Fibrobronchoscopy combined with biopsy can be used to determine the pathology. Pathological examination showed that spindle cell sarcomatoid carcinoma was common, and pleomorphic carcinoma was found in 1 case.^[7]^ Microscopically, the heterotypic spindle cells are mainly arranged in bundles, oval or pleomorphic, and easily observed in mitosis, and immunohistochemical expression of vimetin markers,may be accompanied by squamous epithelial atypia or squamous cell carcinoma components. Here, we report a case of primary sarcomatoid carcinoma of the trachea(PSCT) and analyze its clinicopathological features, immunohistochemistry, treatment, and prognosis.

## 2. Case presentation

**Figure 1.**
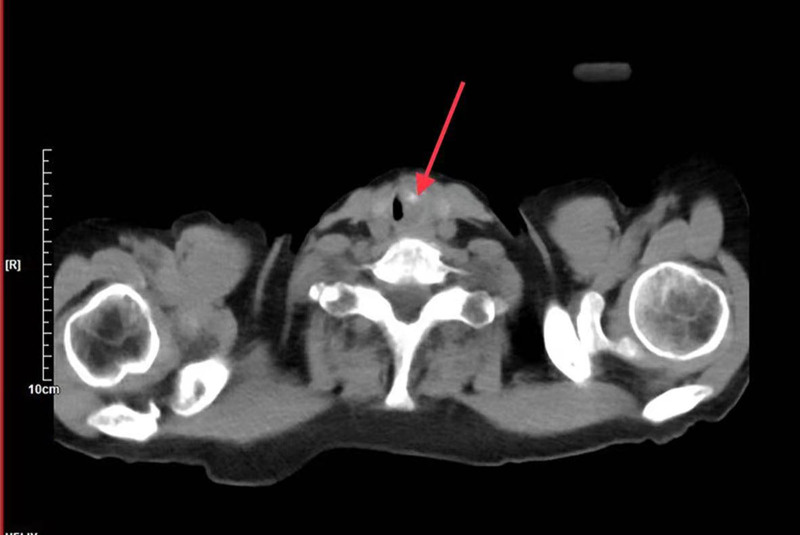
CT examination showed soft tissue shadow with calcification on the left posterior wall of the initial tracheal segment, with local tracheal cavity stenosis accounting for about 80% (red arrow).

A73-year-old female with no history of smoking was admitted to the hospital on August 13, 2020, with shortness of breath for more than 1 month. More than 1 month ago, the patient developed shortness of breath when she was in the right decubitus position, but the left decubitus position improved. During deep inhalation, she heard a high aspiratory laryngeal sound with no difficulty eating, chest tightness, chest pain, nausea, or vomiting. The patient was not affected by life and did not seek medical treatment. Physical examination revealed a pulse of 74 beats/min, breathing of 22 beats/min, blood pressure of 124/65 mm Hg, body temperature of 37°C, clear consciousness, no cyanosis of the lips, no irritation of the jugular vein, no swelling of the superficial cervical lymph nodes, centered trachea, triscuval sign on aspiration, and clear breath sounds in both the lungs. She had a history of radiotherapy for a nasopharyngeal malignant tumor 16 years prior, no recurrence during long-term follow-up, and type II diabetes for several years. CT examination of the neck and chest revealed soft tissue shadow with calcification on the left posterior wall of the initial tracheal segment, 1.6 × 1.0 cm in size and 2.2 cm in length, infiltrating the wall of the trachea near the whole layer, with local tracheal cavity stenosis accounting for about 80% (Fig. 1), and there were no significantly enlarged lymph nodes. Serological examination of the tumor indicated that carcinoembryonic antigen, CA125, squamous cell carcinoma-associated antigen, and alpha-fetoprotein (3rd generation) levels were within the normal ranges. On August 14, 2020, a painless bronchoscopic airway neobiological biopsy was performed, and it was observed that the glottis was well closed, new organisms were present below the glottis, the lumen was narrow, and the mirror barely entered. In this biopsy, the lower trachea was unobstructed, the carina was sharp and centered, and the mucosa was normal.

**Figure 2.**
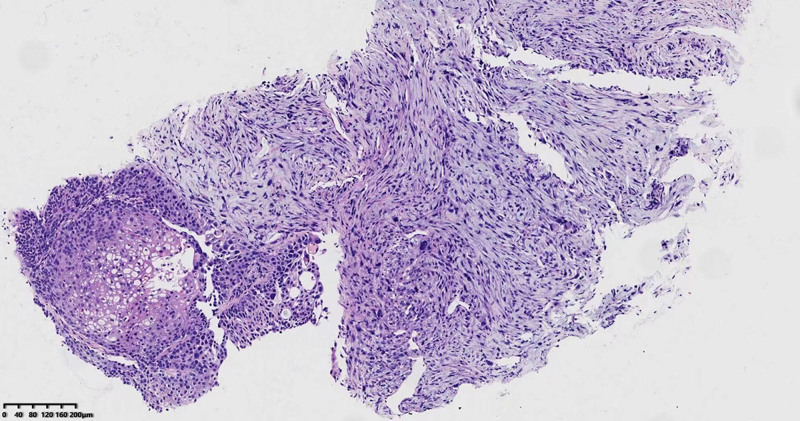
HE staining of the tumor was characterized by a heterogeneous spindle cell and high-grade squamous intraepithelial tumor (HE × 100).

**Figure 3.**
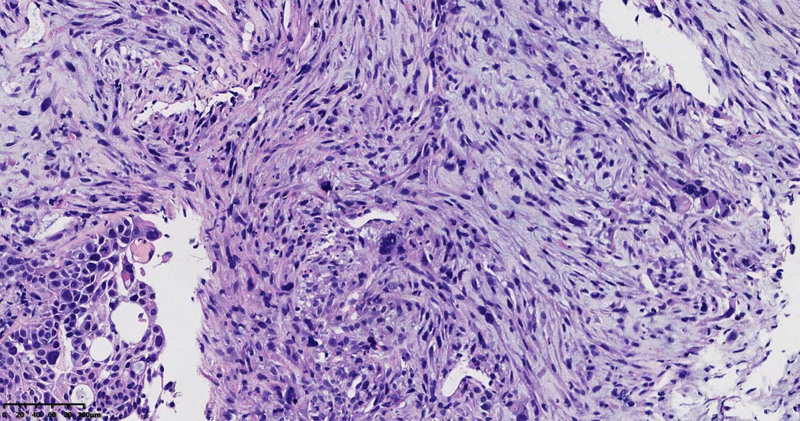
A few fat spindle and pleomorphic cells were scattered, the nuclei were large and deeply stained or irregular (HE × 100).

Microscopic observation. The tissue was fixed with 4% neutral formalin (24 hours at 25°C), embedded in paraffin, and 4‑µm serial sections were prepared and subjected to hematoxylin and eosin (H&E) staining (8 hours at 25°C). At low magnification (digital slice scanner; Ningbo Jiangfeng Biological Information Technology, Co., Ltd.), the tumor was mainly composed of heterotypic spindle cells and a few high-grade squamous intraepithelial tumors became compact (Fig. 2). At high magnification, spindle cells were moderately heterotypic, with 8/10 high-power fields mitoses, and pathological mitosis occurred simultaneously; a few fat spindles and pleomorphic cells were scattered, and the nuclei were large, deeply stained, or irregular (Fig. 3), the cytoplasm was lightly stained and no obvious inflammatory cell infiltration was observed in the interstitium. Some squamous cells had moderate-to-severe atypia in 2/3 layers, large and deeply stained nuclei, and some squamous cells had clear cytoplasm in the central nest-like area. Patchy necrotic foci were observed in the surrounding tissues.

**Figure 4.**
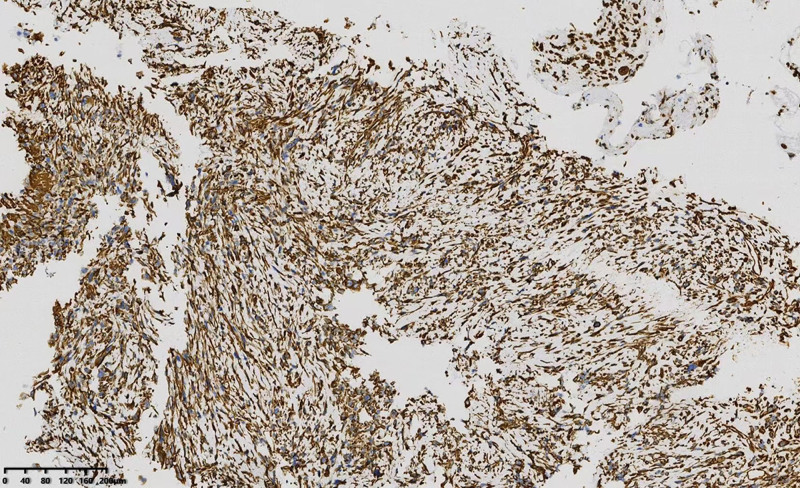
Immunohistochemical staining revealed that the spindle tumor cells were strongly positive for vimentin (× 100).

Immunohistochemical staining with the spindle cell component results were positive for vimentin (Fig. 4), partial positivity for desmin, and a positive result (40%) for the Ki‑67 proliferative index; negative for cytokeratin, epithelial membrane antibody, smooth muscle actin, S‑100 protein, CD34, p63, p40, TTF-1,CK7, Napsin A, CgA, Syn, CD56, Catenin-β, and Calponin; and a small amount of epithelial component results were positive for cytokeratin, epithelial membrane antibodies, p63, and p40.

Pathological diagnosis: Primary sarcomatoid carcinoma of the trachea.

Clinical stage T2N0M0; IIA.

Treatment process: After the diagnosis, a multidisciplinary joint diagnosis and treatment discussion was conducted under the auspices of the respiratory department of a superior hospital. As the patient had a history of head and neck radiotherapy for nasopharyngeal cancer, her general condition was poor, and her body mass index was 16.4, she could not undergo total resection or chemotherapy. At the same time, the patient had obvious shortness of breath, which needed to be treated as soon as possible to improve the clinical symptoms and prolong the life of the patient. Therefore, partial tumor resection was performed under general anesthesia with tracheoscopy followed by local radiotherapy. Gross tumor volume: 60GY/25Fx, planning tumor volume: 50GY/25Fx, and radiotherapy was performed 20 times, with sudden shortness of breath. CT of the neck revealed a local tracheal stenosis involving the subglottis. Consultation with the otolaryngology department was requested, and emergency tracheotomy was performed on October 9, 2019. As the tumor could not be completely cured, the patient experienced repeated coughing and shortness of breath and died of the disease 15 months later.

## 3. Discussion

The incidence of primary tracheal cancer is approximately 0.1/100,000 people per year. In 2011, Urdaneta et al found that the male-to-female ratio of this tumor was 11:9 (578 patients in total). The main histological type was squamous cell carcinoma (45%), while other histological types included adenoid cystic carcinoma and neuroendocrine carcinoma.^[2]^ TSC is included in the World Health Organization classification of thoracic tumors (5th edition) in 5 subtypes: pleomorphic, giant cell, spindle cell, pulmonary blastoma, and carcinosarcoma.^[9]^ Since Aksu et al first reported PSCT in 2009, only 6 cases have been reported in the literature (Table 1). This article reports a case and analyzes its clinicopathological, immunohistochemical, and treatment prognoses based on the literature.

**Table 1:** Clinicopathologic characteristics on the 6 literature cases.

Author/reference number	Age/sex	Smoker (yes/no)	Site	Size (cm)/appearance/blocking degree	Symptom	Therapy	Clinical outcome (mo)	Clinical stage
Aksu et al^[3]^	78/M	Y	Thoracic trachea	N.M/exophytic, obstructing the left and right bronchi by 90% and 75%	Progressive dyspnea, cough for 3 months, weight loss of 2 kg	CT (cisplatin-based)	DOD after 5 mo	N.M
Gurria et al^[4]^	75/M	Y	Cervical trachea	1.5/exogenous/50% obstruction of trachea	8 d of progressive dyspnea, wheezing, and productive cough	Trachea sleeve resection anastomosis	Alive at 6 mo, no recurrence	T1aN0M0
Jang et al^[5]^	37/M	N	Cervical trachea	2.2/polypoid/partial obstruction of trachea	1 mo of bloody sputum, cough, and foreign body sensation in the neck 3 kg weight loss over 3 mo, dyspnea and loss of appetite	Segmental tracheal resection with anastomosis, CT (docetaxel-based and carboplatin-based)	Alive at 60 mo, no recurrence	N.M
Sokoya et al^[6]^	78/F	N.M	Cervical trachea	Intraluminal mass invades thyroid gland	Large neck mass, hoarse voice (time frame not specified)	Surgery: total thyroidectomy, tracheal sleeve resection and anastomosis, central and right neck dissections, CT(carboplatin-based), radioactive iodine	N.M	T4aN1M0
Saikawa et al^[7]^	66/M	Y	Cervical trachea	1.3/exophytic/obstructed trachea	Difficulty breathing and wheezing for about a month	Tracheotomy, RT	2 mo still alive	N.M
Moore et al^[8]^	70/F	Y	Cervical trachea	N.M/ exophytic thyroid invasion/trachea obstruction 80%	10 days of sore throat, dyspnea, and stridor	Debulking of tumor Planned for stent	DOD after 4.5 mo	N.M
Present	73/F	N	Cervical trachea	1.6/exophytic/trachea obstruction 80%	Shortness of breath more than a month	Partial tumor resection under tracheoscope, RT	DOD after 15 mo	T2N0M0

Abbreviations: DOD = died of disease, F = female, M = male, N.M. = Not mentioned; RT and CT = radiotherapy and chemotherapy.

Clinical features: patients with PSCT ranged in age from 37 to 78 years, with an average age of 68.1 years, and a male-to-female ratio of 4:3. The ratio of the cervical to the thoracic tracheal region was 6:1. Clinical symptoms are mainly dyspnea or accompanied by cough, sputum, wheezing, blood in sputum, emaciation, and hoarseness. Similar to asthma and chronic obstructive pulmonary disease, a neck mass can be misdiagnosed as a thyroid tumor.^[6]^ As the tracheal lumen is usually large, symptoms of obstruction do not appear until the mass is > 50% to 75% of the lumen diameter.^[10]^ CT examination of 7 cases of tracheal tumors showed intraluminal and intraluminal growth obstructing the airway to varying degrees, and 2 cases showed thyroid gland involvement.^[4,6]^ In some cases, endotracheal masses were detected using tracheoscopy and pathological biopsies were obtained. The patient in our case was a nonsmoker who had shortness of breath for more than 1 month. CT revealed soft tissue masses in the upper tracheal segment accompanied by calcification and local tracheal stenosis.

The causes of most subtypes of primary tracheal cancer have not been established, and smoking and exposure to other aerosolized carcinogens, including hydrocarbons, have been identified as potential risk factors.^[11]^ In the PSCT literature, 4 cases had smoking history, 1 case had no smoking history, 1 case was not mentioned. This case had no smoking history also. One case of laryngeal cancer was treated with radiotherapy and chemotherapy 2 years ago.^[7]^ Our patient had no recurrence or metastasis after long-term follow-up with radiotherapy for nasopharyngeal cancer 16 years prior. Although PSCT has a certain relationship with smoking, its relationship with radiotherapy remains to be confirmed.

Pathological features: The neoplasms were exoplastic in appearance and microscopically composed of morphologically varied neoplastic spindle cells arranged in bundles or matriculae, long spindle cells, deeply stained nuclei, and pleomorphic and atypical mitosis. They may be associated with squamous epithelial atypia or squamous epithelial carcinoma (two tumor components closely associated or migrating to each other),^[4,7]^ and may be associated with chronic inflammatory cell infiltration. Thyroid invasion occurred in 2 patients, including 1 with right zone IV lymph node metastasis.^[6]^ Immunohistochemical spindle cells expressed vimentin, EMA, or SMA, but did not express CK, S-100, CD31, Desmin, P40, and 1 case expressed P63.^[5]^ Epithelial components are expressed as epithelial markers. In this case, the pathological findings of spindle cells were closely associated with high-grade squamous intraepithelial tumors, and the spindle cells only expressed vimentin.

Treatment and prognosis: Surgical resection is the first-line treatment for primary tracheal malignancies, and the 5-year survival rate of patients treated with surgical resection is 50%.^[12]^ Adjuvant radiotherapy is recommended for all types of locally advanced tracheal cancers and for patients with potentially positive postoperative margins. In nonsurgical candidates, radiotherapy or radiotherapy combined with platinum-based concurrent chemoradiotherapy is considered the first-line therapy and palliative option for advanced disease.^[13]^ Two cases of TSC were reported in the literature,^[3,8]^ respectively, after 4.5 to 5 months of death due to the disease. One patient had no recurrence or metastasis 60 months after complete surgical resection combined with radiotherapy and cisplatin-based chemotherapy.^[5]^ The other 2 cases^[4,7]^ were followed up for 2 to 6 months without recurrence or metastasis, but the prognosis was still very uncertain due to the short time. The patient died 15 months after the palliative surgery and radiotherapy.

## 4. In conclusion

PSCT is relatively rare, and the clinical symptoms are often dyspnea due to airway obstruction. The diagnosis depends on the pathological diagnosis. At present, there is no uniform treatment plan, usually a combination of surgery-based therapy, radiation and/or chemotherapy, and the patient’s systemic condition. Due to the small number of cases at this site, more clinical data need to be accumulated for further studies.

## Author contributions

**Conceptualization:** Bin Huang.

**Formal analysis:** Junjie Yang.

**Investigation:** Haiyan Ge.

**Methodology:** Mingqi Huang.

**Visualization:** Jialing Xu.

**Writing – original draft:** Xiuwen Yu.

**Writing – review & editing:** Jialing Xu.
